# AI-based clinical prediction model for early infectious disease classification in the emergency department

**DOI:** 10.3389/frai.2026.1894719

**Published:** 2026-08-19

**Authors:** Sara N. Søgaard, Helene Skjøt-Arkil, Christian B. Mogensen, Theis Aagaard, Thomas Kronborg

**Affiliations:** 1 Department of Regional Health Research, University of Southern Denmark, Odense, Denmark; 2 Emergency Department, University Hospital of Southern Denmark, Aabenraa, Denmark; 3 Department of Health Science and Technology, Aalborg University, Gistrup, Denmark

**Keywords:** AI-based infection prediction, artificial intelligence, clinical decision support, clinical prediction, clinical risk stratification using AI, differential diagnosis, emergency department, infection

## Abstract

**Background:**

Diagnosing infections remains challenging. Clinicians rely on scoring systems and experience, but artificial intelligence (AI) is used to support decision-making by integrating clinical data. However, most AI models focus on predicting adverse outcomes (e.g., ICU admission or sepsis) rather than differentiating between infection types.

**Objective:**

To develop predictive models and evaluate their discriminatory performance for classifying infected patients into pneumonia, urinary tract infection (UTI), and other infections, and to assess the incremental value of adding clinical tests to routine variables.

**Methods:**

Six Random Forest models were developed on datasets sharing a set of standard variables but differing in the added clinical variables. The models predicted infection type across three categories using a one-vs-rest framework. Data were divided into 70% for model training and 30% for model testing. The decision threshold was set with sensitivity fixed at 0.8, and specificity, negative predictive value, positive predictive value, and accuracy were reported. Variable importance was assessed, and the top five predictors per model were identified.

**Results:**

The model including standard variables and urine data achieved the highest AUC for UTI, whereas the total model achieved the highest AUC for pneumonia. Overall, urine and total models demonstrated the strongest performance. Key predictors included positive chest X-ray findings, abnormal auscultation, suspected pulmonary disease on imaging, neutrophil count, weight, urine culture, and a leukocyte-positive dipstick.

**Conclusion:**

Machine learning models can potentially improve infection classification at ED admission when routine data are supplemented with relevant diagnostics. Optimal combinations of clinically relevant data markedly enhance diagnostic performance.

## Highlights

- Random forest model can potentially classify infection types at emergency department admission using routine clinical data.
- Adding targeted diagnostic tests, especially urine-based variables, improves model performance in distinguishing between infectious disease categories.
- Key predictors include radiographic signs of pneumonia, abnormal lung auscultation, inflammatory markers and urine test results.

## 1 Introduction

Infections remain one of the primary causes of hospital admissions through emergency departments (EDs), and timely identification and initiation of targeted antibiotic treatment are essential to achieving optimal patient outcomes and responsible antibiotic consumption (Filbin et al., 2014; Nassisi and Oishi, 2012).

Pneumonia and urinary tract infections (UTIs) are among the most frequent infectious presentations in the emergency department (Kennedy et al., 2010). However, diagnosing both conditions in the ED is challenging, as symptoms are often mild or non-specific, and available methods for focal and etiological diagnosis have limited sensitivity and specificity, with many test results becoming available only after decisions regarding antibiotic treatment have been made (Caterino et al., 2019). Improving diagnostic accuracy in this context therefore requires a multifaceted approach that integrates clinical expertise with modern advanced technologies.

Within diagnostics, artificial intelligence (AI) has gained significant traction across multiple clinical domains (Adachi et al., 2024; Ngombu et al., 2023; Chadaga et al., 2023), and an increasing number of decision-support tools are being developed to streamline clinical workflows and augment clinical decision-making (Nicolaou et al., 2024). A central advantage of AI and machine learning (ML) in clinical practice is their ability to identify complex patterns in large datasets that may not be readily apparent to clinicians (Yuan et al., 2020). These datasets include both structured information such as laboratory values and vital signs, and unstructured data including free-text documentation in electronic health records (Horng et al., 2017). Studies have demonstrated that ML models incorporating free-text input, such as clinical guidelines, can improve diagnostic performance and support clinicians in challenging diagnostic tasks (Wang et al., 2023).

A variety of approaches have been proposed to detect infections in the ED; however, most existing models primarily focus on identifying sepsis rather than infection itself (Chaganti et al., 2025). In parallel, healthcare systems are increasingly adopting AI- and ML-based tools to support clinical decision-making across specialties (Currey and Tarabichi, 2025; Yao et al., 2025; Campbell et al., 2025). Despite this progress, the use of AI for early detection and classification of infections—prior to progression to sepsis—remains limited. Much of the current literature centres on sepsis prediction, implicitly assuming that infection has already advanced to a severe and systematic stage. This creates a critical gap in early diagnostic efforts, where timely identification of the presence and type of infection, before the onset of sepsis, remains clinically important and potentially lifesaving.

We have previously developed an AI-model designed to predict infection among patients presented to the ED. This model served as a rule-in/rule-out system, distinguishing between the presence versus absence of infection (Søgaard et al., 2026).

In this study, we aimed to develop a predictive model capable of classifying patients already classified as having an infection into three clinically relevant infectious disease categories: pneumonia, urinary tract infections (UTIs), and other infections. We sought to examine how the incremental addition of various clinical tests and assessments to a baseline set of routinely collected variables, influenced the model’s predictive performance. Furthermore, a central focus of the study was to identify and explain the most informative predictors distinguishing the infectious disease categories at the time of ED admission.

## 2 Methods

The study is reported according to Transparent Reporting of a multivariable prediction model for Individual Prognosis or Diagnosis (TRIPOD) statement including the update for artificial intelligence (Collins et al., 2024).

### 2.1 Data

Data in this study was based on the clinical predictive modelling study of data collected prospectively in a multicentre, multifaceted study – the INDEED study (Trial IDs: NCT04681963, NCT04667195, NCT04652167, NCT04686318, NCT04686292, NCT04651712, NCT04645030). The INDEED study protocol has been previously published (Skjøt-Arkil et al., 2021).

### 2.2 Patients

The present study is based on 966 patients initially included in the INDEED study and included patients aged 18 years or older who were admitted to the ED suspected of having an infection and were able to provide consent. Patients were excluded if they were transferred immediately to the intensive care unit (ICU), if participation could delay lifesaving treatment, if they had been admitted within the last 14 days, if they had a confirmed COVID-19 infection within the previous 14 days, or had severe immunodeficiency. Patients in the study were admitted to the EDs at Odense University Hospital, Hospital Lillebælt in Kolding, and Hospital Sønderjylland in Sønderborg and Aabenraa between March 1, 2021, and February 28, 2022.

The final diagnosis was established by an expert panel at each participating hospital—Odense University Hospital, Hospital Lillebælt, and Hospital Sønderjylland—comprising a specialist in emergency medicine and a specialist in infectious diseases, both with extensive experience in the management of acute infections. In total, eight experts participated in the study. Diagnoses were assigned based on comprehensive chart review, incorporating all available clinical information obtained within the first week of admission, including routine laboratory results and imaging findings. No predefined checklists or formal diagnostic criteria were applied. In cases of disagreement, the final diagnosis was reached through consensus between the two experts.

**Figure 1:**
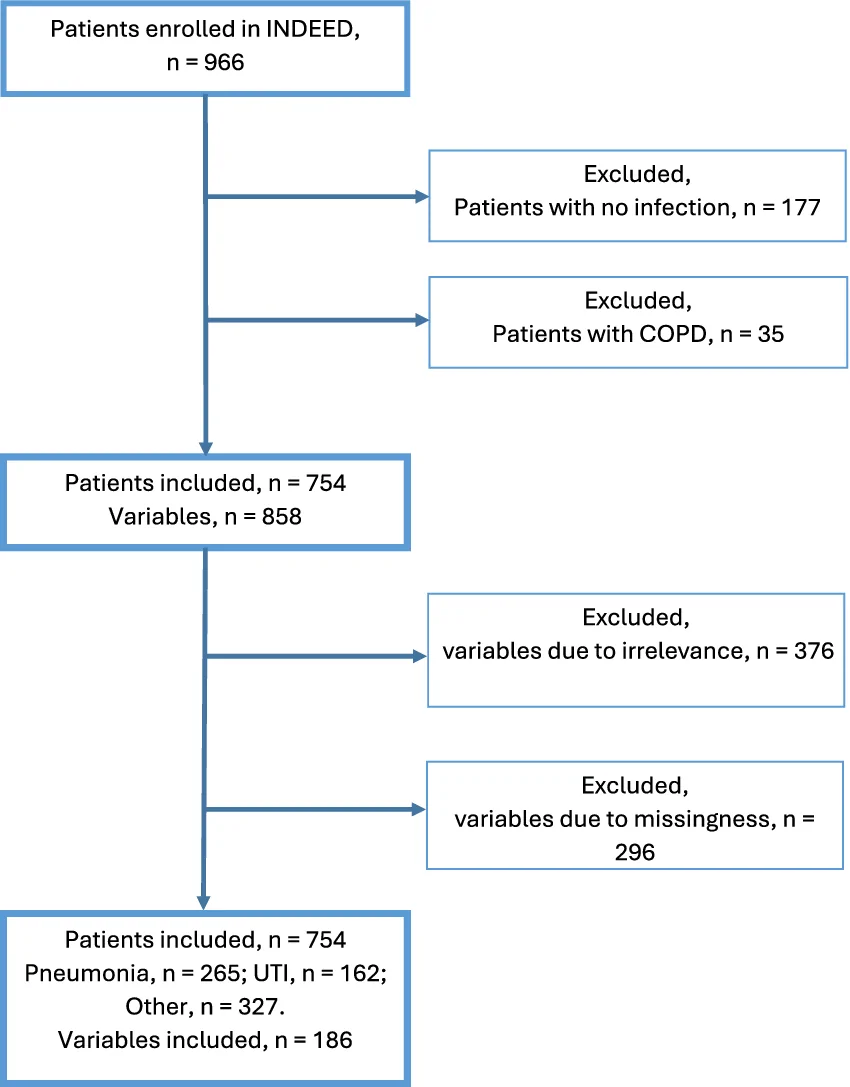
Flowchart of patient and variable inclusion in an AI-based model predicting pneumonia, urinary tract infection, or other infections in patients with infections seen in emergency departments in Southern Denmark, 2021–2022. INDEED: infectious diseases in emergency departments (Skjøt-Arkil et al., 2024); COPD: chronic obstructive pulmonary disease.

In the analysis in our study, we further excluded patients with no verified infection, and patients with chronic obstructive pulmonary disease (COPD) and no secondary infection. An overview of patient inclusion can be found in Figure 1.

### 2.3 Data preparation, missing data, and variables

The INDEED dataset included 858 variables extracted from medical records, including, among others, variables such as clinical findings, patient-reported symptoms, antibiotic prescriptions prior to hospital admission, and triage category (Plesner et al., 2015). The dataset also included results from blood tests, diagnostic imaging, and microbiological analysis of blood and sputum samples.

Prior to analysis, the dataset was reviewed and preprocessed. Variables with more than 10% missing data were excluded. For the remaining variables, missing values were imputed using mean imputation prior to model development. In addition, 376 variables deemed irrelevant to the objectives of the present study were removed. These included time-dependent variables (e.g., time of admission, blood sample collection, CT ordering, and CT/X-ray acquisition), antibiotic prescription data, and readmission data. An additional 296 variables were excluded due to excessive missingness (>10%), resulting in the removal of 672 variables in total. Furthermore, highly detailed descriptors of CT and ultra-low-dose CT (ULD-CT) imaging, comprising more than 200 variables, were manually reviewed and consolidated into broader CT and ULD-CT categories representing clinically relevant imaging characteristics. This approach reduced unnecessary model complexity while preserving relevant imaging information.

To classify patients into three infectious disease categories while investigating the contribution from various clinical tests and assessments, we developed prediction models based on six separate datasets.

The initial prediction model was based on a dataset consisting of standard variables (SV) – i.e. the initial clinical assessment a list of the SV can be found in Appendix 1. This SV dataset was defined to reflect information available at the time of initial emergency department assessment within 4 hours of admission. According to regional clinical assessment guidelines, patients must have a treatment plan within 4 h of admission to the ED, why this time point was chosen.

We developed five extended models with a focus on clinical applicability and incremental diagnostic value. Importantly, these variables were not limited to information available at the time of presentation but reflected investigations obtainable during the predefined ED admission period of up to 4 h. With the exception of urine culture results, all additional features can realistically be available within this time window and therefore represent clinically relevant information that could be incorporated into the initial ED assessment. The extended models were included to assess their incremental contribution to diagnostic performance and to identify investigations that may warrant earlier prioritization in the diagnostic pathway. Each data set contained the following types of variables (The complete list of variables can be found in Appendix 2):

1. SV: Routinely available clinical, demographic, and laboratory data within the first 4 hours of ED admission.
2. Bio: SV and infection biomarkers Club Cell protein 16 (CC16), Interleukin 6 (IL6), Procalcitonin (PCT), soluble urokinase Plasminogen Activator Receptor (Supar) and Chitinase-3-like protein 1 (YKL40).
3. CT: SV and radiological findings from CT and ULD CT.
4. POC: SV and respiratory-specific biomarkers.
5. Urine: SV and urine dipstick analyses for glucose, ketones, blood, protein, nitrite, leucocytes, and urine culture for bacteria.
6. Total: Complete dataset.

Based on results from a previous study comparing four algorithms for predicting infection in the emergency department (Søgaard et al., 2026), we selected the random forest approach for the present study. Infection status in the current analysis was, however, determined by the expert panel diagnosis rather than by application of the previously developed infection detection model. Therefore, validation of the complete sequential workflow (infection detection followed by infection classification) remains to be performed.

### 2.4 Outcome

The primary outcome of this study was the classification of patients in the infectious disease categories: pneumonia, urinary tract infection (UTI), or other infection. The predicted outcome was compared to the final diagnosis as determined by the expert panel.

### 2.5 Model training and validation

The data was divided into 70% for model training and 30% for model testing. The training data was used to train six Random Forest (RF) models based on each of the data sets with a random state of 42 and 100 estimators. Prior to model training, numerical variables were standardized to ensure consistent processing across the evaluated machine learning models. No class weighting, resampling, or other class balancing approaches were applied, and all models were trained using the original class distribution. Hyperparameters, including the minimum samples per leaf and maximum tree depth, were optimized using a grid search with five-fold cross-validation performed within the training set.

The test set was used to test the model performance on previously unseen data, using the optimal hyperparameters identified during the grid search, where we reported the area under the receiver operating characteristics curve (AUC). Furthermore, the decision threshold corresponding to a sensitivity of approximately 0.80 was selected using the ROC curve on the independent test set by identifying the first threshold achieving sensitivity ≥0.80. The corresponding specificity, negative predictive value (NPV), positive predictive value (PPV), and accuracy were then calculated based on this threshold. Calibration curves were generated for the two best-performing models to assess the agreement between predicted probabilities and observed outcomes. The calibration curves were evaluated for each infectious disease category and are presented in the Supplementary data. The threshold was set to ensure comparison across the models and prioritise correct identification of true positive cases and reduce the risk of false negatives in clinical classification.

Furthermore, feature importance was evaluated, and class-specific feature effect interpretations were conducted using the trained RF models. For each predictor, class-wise feature distributions were compared against the remaining classes to evaluate directionality of association within each outcome category. Features with consistently higher values within a given class were interpreted as positively associated with that class, whereas features with consistently lower values were interpreted as negatively associated. This directional interpretation reflects relative differences in feature distributions between classes and does not imply causality but provides additional interpretability by describing how predictors differentiate between infectious disease categories within the models. Data pre-processing was performed in Stata Statistical Software (Release 17, StataCorp LLC, College Station, TX, USA) and analyses were performed in python (version 3.7) with relevant packages.

## 3 Results

### 3.1 Participants

There were 966 patients in the INDEED study, and we excluded patients with no infection (*n* = 177) and patients with COPD (*n* = 35), resulting in a total patient inclusion of 754 patients (Figure 1). The distribution of infection types among the included patients was as follows: 35.1% had pneumonia (*n* = 265), 21.5% had UTI (*n* = 162), and 43.4% had other infections (*n* = 327,). Characteristics for the patients at baseline are presented in Table 1.

**Table 1:** Baseline characteristics of 754 emergency department patients in Southern Denmark (2021–2022) included in an AI-based model predicting pneumonia, urinary tract infection, and other infections.

Characteristic	Pneumonia	UTI	Other	Total
Age in years, mean (SD)	71.2 (15.8)	71.6 (15.9)	64.7 (19.2)	68.9 (17.4)
Male sex, n (%)	145 (34.3)	116 (27.4)	162 (38.3)	423 (56.1)
Medication				
Polypharmacy (≥5 drugs), *n* (%)	171 (40.1)	114 (26.8)	141 (33.1)	426 (56.5)
Vital signs				
Heart rate in beats/min, mean (SD)	93 (19)	91 (18)	88 (18)	91 (18)
Respiratory frequency, n/min (SD)	22 (6)	19 (4)	19 (4)	20 (5)
Blood oxygen saturation, % (SD)	94 (4)	97 (3)	96 (2)	96 (3)
Systolic blood pressure, mmHg (SD)	134 (21)	128 (23)	132 (22)	132 (22)
Diastolic blood pressure, mmHg (SD)	74 (14)	72 (16)	75 (15)	74 (15)
Temperature, °C (SD)	37.6 (1)	37.8 (1)	37.4 (1)	37.5 (1)
Clinical findings				
Crepitations on auscultation, *n* (%)	126 (67.7)	32 (17.2)	28 (15.1)	186 (24.7)
Ronchi on auscultation, *n* (%)	44 (80)	4 (7.3)	7 (12.7)	55 (7.3)
Prolonged expiratory phase on auscultation, *n* (%)	19 (73.1)	2 (7.8)	5 (19.2)	26 (3.4)
Diminished breath sounds on auscultation, *n* (%)	16 (84.2)	0 (0)	3 (15.8)	19 (2.5)
Pain on abdominal palpation, *n* (%)	40 (25)	63 (39.4)	57 (35.6)	160 (21.2)
Triage, *n* (%)*				
Red/orange	92 (50)	41 (22.3)	51 (27.7)	184 (24.4)
Yellow	132 (34.3)	113 (29.4)	140 (36.4)	385 (51.2)
Green/blue	53 (28.7)	45 (24.3)	87 (47.0)	185 (24.5)
Blood samples				
Haemoglobin, mmol/L (SD)	7.9 (1.1)	7.8 (1.1)	8.1 (1.2)	7.9 (1.1)
Leucocytes, ×10^9^/L (SD)	13.6 (7.2)	13.7 (6.2)	11.7 (5.6)	12.9 (6.4)
C-reactive protein, mg/L (SD)	139.6 (103)	147.1 (98)	128 (103)	137.3 (102)
Creatinine, μmol/L (SD)	92.9 (49.6)	118.2 (70.4)	116.1 (117.9)	108.1 (86.3)
Sodium, mmol/L (SD)	136.3 (4.3)	135.9 (4.0)	136.2 (4.2)	136.1 (4.2)

SD: Standard deviation.

*Triage: Danish emergency process triage (Plesner et al., 2015).

### 3.2 Model development

From the original 858 variables, 376 were excluded due to no relevance for the model and 296 were excluded due to missing data (Figure 1). The SV dataset contained 112 variables, whereas the Bio dataset contained an additional 7 variables, CT dataset an additional 6 variables, POC dataset an additional 34 variables and Urine dataset an additional 8 variables. The complete dataset included all of the mentioned variables, as well as additional standard culture samples from the respiratory tract, blood, and urine, resulting in a total of 186 variables.

### 3.3 Model performance

**Figure 2:**
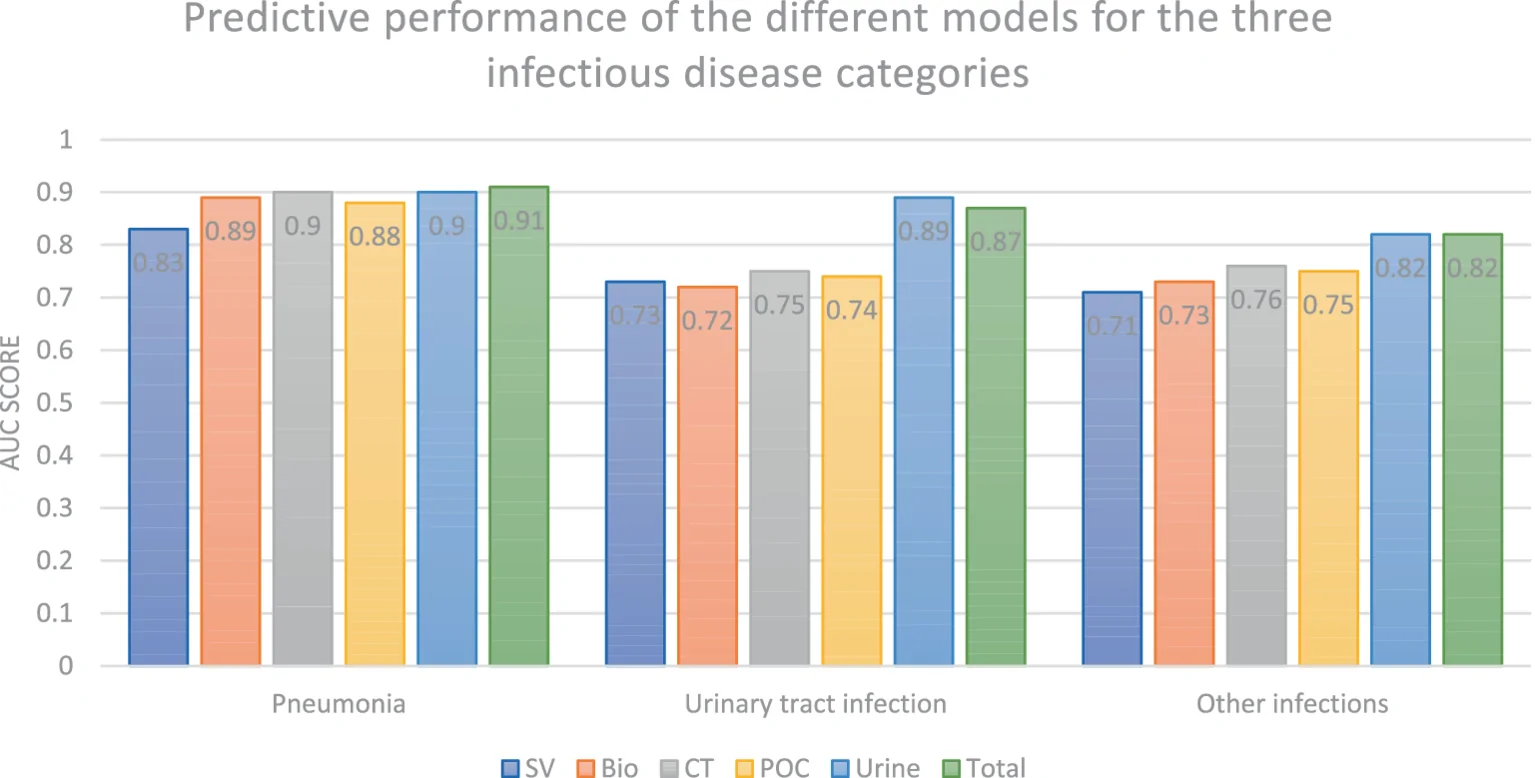
Comparative predictive performance of AI-based models for pneumonia, urinary tract infection, and other infections in emergency department patients in Southern Denmark (2021–2022). AUC, area under the curve; SV, standard variables, Bio: SV + biomarkers, CT: SV + radiological imaging, POC: SV + POC respiratory analysis, Urine: SV + urine analyses, Total: all variables.

Model performance was evaluated within a one-vs-rest framework for the three infectious disease categories (Table 2 and Figure 2). For each category, performance reflected the ability of the models to discriminate patients belonging to the target infection category from patients belonging to the remaining infection categories. Across all six evaluated models, a sensitivity of at least 0.80 was achieved. However, this resulted in considerable variability in specificity, particularly for the UTI discrimination, where baseline SV alone performed poorly (specificity 0.51–0.69).

**Table 2:** Performance metrics of AI-based models for predicting pneumonia, urinary tract infection, and ER infections in emergency department patients.

Model	Sensitivity, %	Specificity, %	Negative predictive value, %	Positive predictive value, %	Accuracy, %	AUC for the model (CI 95%)
SV	P: 0.80 U: 0.81 O: 0.81	P: 0.69 U: 0.51 O: 0.51	P: 0.86 U: 0.89 O: 0.81	P: 0.58 U: 0.37 O: 0.51	P: 0.72 U: 0.59 O: 0.63	P: 0.83 (0.77–0.88) U: 0.73 (0.65–0.81) O: 0.71 (0.65–0.77)
Bio	P: 0.80 U: 0.81 O: 0.81	P: 0.80 U: 0.43 O: 0.57	P: 0.88 U: 0.87 O: 0.82	P: 0.69 U: 0.34 O: 0.54	P: 0.80 U: 0.53 O: 0.66	P: 0.89 (0.84–0.93) U: 0.72 (0.64–0.79) O: 0.73 (0.67–0.79)
CT	P: 0.80 U: 0.81 O: 0.82	P: 0.88 U: 0.48 O: 0.60	P: 0.89 U: 0.88 O: 0.84	P: 0.79 U: 0.36 O: 0.56	P: 0.85 U: 0.57 O: 0.68	P: 0.90 (0.86–0.94) U: 0.75 (0.68–0.82) O: 0.76 (0.69–0.82)
POC	P: 0.80 U: 0.83 O: 0.83	P: 0.86 U: 0.51 O: 0.62	P: 0.89 U: 0.90 O: 0.85	P: 0.76 U: 0.37 O: 0.58	P: 0.84 U: 0.60 O: 0.70	P: 0.88 (0.83–0.93) U: 0.74 (0.68–0.82) O: 0.75 (0.69–0.81)
Urine	P: 0.80 U: 0.81 O: 0.82	P: 0.90 U: 0.84 O: 0.73	P: 0.89 U: 0.93 O: 0.86	P: 0.81 U: 0.64 O: 0.65	P: 0.86 U: 0.83 O: 0.76	P: 0.90 (0.86–0.94) U: 0.89 (0.84–0.93) O: 0.82 (0.76–0.87)
Total	P: 0.81 U: 0.81 O: 0.82	P: 0.88 U: 0.79 O: 0.71	P: 0.90 U: 0.92 O: 0.86	P: 0.78 U: 0.57 O: 0.64	P: 0.85 U: 0.79 O: 0.75	P: 0.91 (0.87–0.95) U: 0.87 (0.81–0.92) O: 0.82 (0.76–0.87)

P: pneumonia, U: urinary tract infection, O: Other infection.

SV: standard variables, bio: SV + biomarkers variables, CT: SV + radiological imaging, POC: SV + POC respiratory analysis, Urine: SV + urine analyses, total: all variables.

The addition of Bio, CT, POC, and Urine variables improved model performance to varying degrees. The most pronounced improvements in specificity were observed for the Urine model, particularly for discrimination of UTI from the remaining infection categories, where specificity increased to 0.84. Similarly, the Total model showed improved performance compared with SV alone, with specificity reaching 0.79 for UTI and 0.71 for other infections.

The other performance metrics followed a similar pattern. NPVs were generally high across all models, especially for UTI (0.87–0.93). PPVs were highest for the pneumonia discrimination (0.58–0.81), but consistently lower for the UTI discrimination (0.34–0.64). Accuracy improved with the addition of clinical variables, with the largest improvement observed for UTI when comparing SV alone (0.59) with the Urine model (0.83) and the Total model (0.79).

When comparing AUC across the one-vs-rest models, the highest discrimination was observed in the Total model and the Urine model for pneumonia (AUC 0.91 and 0.90, respectively). For discrimination of UTI from the remaining infection categories, the Urine model achieved the highest AUC (0.89), followed by the Total model (0.87). For discrimination of other infections from pneumonia and UTI, the Urine and Total models both achieved an AUC of 0.82, indicating improved discrimination compared with the SV model (AUC 0.71).

The total model demonstrated improved overall performance compared with SV alone, particularly in terms of discrimination and accuracy.

Overall, the addition of clinical and diagnostic variables improved discriminatory performance across the evaluated one-vs-rest models compared with SV alone. The Urine model provided the most consistent improvement, particularly for UTI discrimination, where specificity, accuracy, PPV, and AUC were substantially higher compared with the baseline SV model. The Total model showed similar overall performance, with slightly improved discrimination for pneumonia and comparable performance for the remaining infection categories. Calibration curves for the Urine and Total models, the two best-performing models, across the three infectious disease categories are presented in the supplementary data.

### 3.4 Identification of the most informative predictors

**Figure 3:**
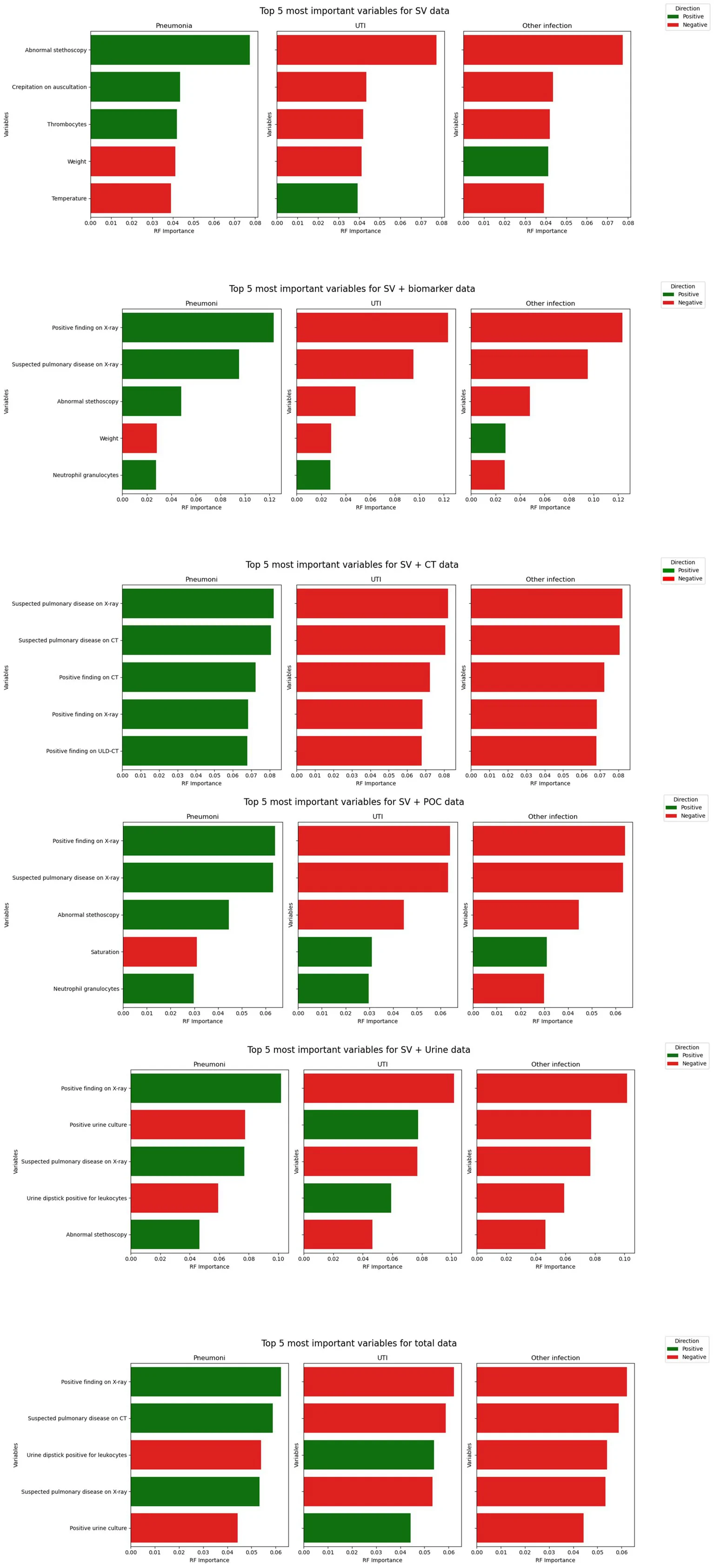
Top five most important predictive variables across six evaluated AI models for pneumonia, urinary tract infection, and other infections in patients in the emergency department. SV, standard variables; UTI, urinary tract infection; RF, random forest; CT, computed tomography; ULD-CT, ultra-low-dose computed tomography; POC, point of care.

Figure 3 shows the top five most informative and predictive variables for all models for the three infectious disease categories. It illustrates both the relative importance of each variable and differences in feature values across diagnostic classes rather than the direction of causal influence on the outcome. Higher or lower values of variables were observed in specific diagnostic classes compared with the remaining classes. For example, an abnormal finding on auscultation was more frequently observed in patients classified as pneumonia compared with urinary tract infection and other infections, whereas the same finding was less frequently observed in the latter two groups.

One of the most dominant variables was a positive finding on chest X-ray, which appeared in all models except the SV model. Two variables were present in four models: abnormal auscultation (SV, SV + biomarkers, SV + POC, SV + urine) and possible pulmonary disease diagnosed on chest X-ray (SV + biomarkers, SV + CT, SV + POC, SV + urine). Finally, four variables were found in two models: neutrophilic granulocytes, weight, urine culture, and urine dipstick positive for leukocytes.

## 4 Discussion

In this study, we evaluated the discriminatory performance of machine learning models for classifying patients, already classified as having an infection, into three clinically relevant infectious disease categories, using a one-vs-rest approach and standard clinical variables supplemented with targeted diagnostic data. We further assessed the incremental diagnostic value of these variables and identified several informative predictors that consistently contributed to model performance across the evaluated models. Dividing the variables into six different datasets based on their clinical information, allowed us to explore the incremental value of each dataset on model performance. The incremental value of adding variables has previous been shown to improve model performance in other research areas or research questions (Khorsand et al., 2025; Enayati et al., 2025).

Importantly, the primary aim of the SV model was to reflect information available at the time of initial ED assessment (within 4 hours of admission), whereas the extended models were included to evaluate the incremental diagnostic contribution of supplementary investigations during the early diagnostic work-up. This approach allowed us to assess which diagnostic modalities provide meaningful added value for classification and may therefore merit prioritisation in early clinical decision-making pathways, rather than implying that all predictors are available at the time of ED presentation.

Thus, the extended models should be interpreted as an evaluation of the potential incremental contribution of additional diagnostic information during the early clinical pathway, rather than as models restricted to the initial ED assessment. The subsequent variable importance analysis further illustrates which clinical assessments and diagnostic investigations contributed most consistently across models.

Across all models, sensitivity was pre-set to a minimum of 0.80, this was done to secure a robust ability to identify the infectious disease categories. However, specificity in the six models showed considerable variability, especially for UTI classification when only SV data were used (specificity 0.51–0.69), highlighting the limitations of relying solely on standard clinical information, in this specific infectious disease category. This is supported by previous studies showing that up to one third of patients with UTIs are misdiagnosed when the diagnosis relies solely on clinical criteria without incorporating diagnostic criteria or confirmatory tests (Schmiemann et al., 2010). However, simply including all available data does not necessarily represent the optimal approach. Although the Total model demonstrated improved performance compared with the SV model, particularly with higher AUC values and accuracy, the Urine model achieved comparable or superior performance for UTI prediction. This suggests that adding more diagnostic variables is not inherently associated with better model performance; rather, careful selection of clinically relevant variables may provide a more efficient approach for optimizing predictive performance in a clinical setting.

The inclusion of additional datasets—particularly urine analyses—markedly improved specificity for UTI (up to 0.84), demonstrating the value of targeted diagnostic test in enhancing model discrimination. Diagnostic accuracy have been shown to improve, by adding urine analysis such as urine dipstick (Giesen et al., 2010), however a recent study have concluded that urine dipstick have a limited diagnostic value compared to a symptom driven approach combined with a positive urine culture (Kristensen et al., 2025). These findings rely on a population admitted to an ED, where urine dipstick were performed on almost half of the admitted patients, however only one-fourth presented with symptoms of an UTI. In our study 58 % had a positive urine dipstick result (showing the present of glucose, blood, ketones, leukocytes, protein, or nitrite) consistent with the observations from the aforementioned study.

Similar patterns were observed for other performance metrics: NPVs were generally high across all infection types, while PPVs varied by infection type, with pneumonia predictions being more reliable than UTI. Overall accuracy improved with the addition of CT, POC, or urine data, with urine providing the largest gain for UTI classification (from 0.53 to 0.83) once again highlighting the incremental value of targeted diagnostic data in complementing standard clinical variables.

Evaluation of model discrimination using AUC further supported these findings. The highest AUC values were observed for the Total model for pneumonia (0.91), while the Urine model achieved the highest AUC for UTI prediction (0.89). For other infections, both the Urine and Total models demonstrated comparable performance (AUC 0.82). These findings indicate that targeted inclusion of clinically relevant variables, particularly urine data, can substantially improve model performance for challenging classifications such as UTI. However, incorporating all available variables in the Total model also provided strong overall discrimination, highlighting the importance of balancing data availability with clinically meaningful variable selection.

Analysis of variable importance revealed consistent predictors across models. Notably, a positive chest X-ray finding emerged as one of the most influential variables, being included in all models except the SV-only model. Abnormal auscultation findings and possible pulmonary disease on X-ray were recurrent in multiple models, reflecting their clinical relevance across infection types. Variables such as neutrophilic granulocytes, weight, urine culture, and urine dipstick for leukocytes were also informative, particularly for UTI and combined datasets. Importantly, the directionality of predictors varied by infection type—for example, abnormal auscultation was positively associated with pneumonia but negatively associated with UTI and other infections—highlighting the nuanced relationships between clinical findings and infection outcomes.

Antimicrobial stewardship is commonly described according to the five Ds: the right Diagnosis, Drug, Dose, Duration, and De-escalation (Pulia et al., 2018). While all five components contribute to appropriate antibiotic use, the first step—establishing the correct diagnosis—is fundamental, as it guides all subsequent treatment decisions. In the present study, all patients had a confirmed infection, and the objective was therefore not to distinguish infected from non-infected patients, but rather to classify the most likely infection source at an early stage of the diagnostic process. A RF model may support this diagnostic step by identifying which clinical, laboratory, and diagnostic parameters contribute most to the differentiation between pneumonia, UTI, and other infections. By quantifying the relative importance of routinely available variables, the model may help prioritize the acquisition of diagnostic information that has the greatest impact on early classification and, consequently, on empirical treatment decisions. Improved early classification of the infection source could assist clinicians in selecting more appropriate empirical antibiotic therapy, thereby supporting the diagnostic component of antimicrobial stewardship. Nevertheless, the model should be regarded as a decision-support tool that complements, rather than replaces, clinical assessment and subsequent diagnostic investigations.

Overall, these results emphasize the importance of integrating clinically relevant data modalities to improve predictive performance in infection classification. While standard clinical variables provide a solid baseline, the addition of specific biomarkers, imaging, and urine data can enhance model specificity, accuracy, and discrimination, particularly for infections that are clinically challenging to diagnose. However, the findings also indicate that the optimal combination of variables may be more important than simply increasing the amount of available data. These results support the potential utility of machine learning approaches for guiding more precise and individualized infection diagnostics in hospitalized patients, although further validation studies are needed.

## 5 Limitations

This study included only patients who were able to provide both oral and written informed consent. Consequently, individuals unable to consent—such as those with impaired consciousness, language barriers, or cognitive impairment—were excluded. This selection criterion may have led to the underrepresentation of clinically important subgroups in the development of the model. These populations often present with greater diagnostic complexity and may derive particular benefit from enhanced clinical decision support systems.

A limitation of this study is that the model was evaluated using a one-vs-rest approach, and no decision rule was developed to translate the class-specific probability thresholds into a final patient-level multiclass classification. The development and validation of such a decision strategy are necessary before clinical implementation.

Furthermore, the reference diagnosis was established using all available clinical information obtained during the first week of admission. Therefore, some overlap between predictors included in the extended models and information contributing to the final diagnostic assessment cannot be excluded. This may have introduced incorporation bias and should be considered when interpreting the performance of models incorporating diagnostic investigations obtained during the early clinical pathway.

Although variables were excluded based on predefined criteria (clinical relevance and proportion of missing data), mean imputation and standardization were performed prior to the train–test split rather than exclusively within the training data. This approach may have introduced a small degree of data leakage, potentially leading to a slight overestimation of model performance. However, given the very low proportion of missing data in the final dataset (0.03%), the impact is expected to be limited. Future studies should perform all preprocessing steps involving the data distribution exclusively within the training data before external validation.

In addition, the decision threshold corresponding to a sensitivity of 0.8 was selected using the independent test set rather than exclusively within the training data, which may introduce optimistic bias in the reported performance estimates. Future studies should define classification thresholds within the training data or cross-validation to ensure fully independent model evaluation.

Collectively, these methodological choices represent a limitation of the current study, as parts of the model development pipeline were informed by information outside the training data and may have contributed to slightly optimistic performance estimates.

To improve generalisability, future studies should externally validate the model in larger emergency department cohorts that reflect the full range of clinical presentations.

## 6 Conclusion

This study shows that machine learning models have the potential to improve classification of infectious diseases at ED admission when standard clinical variables are supplemented with targeted diagnostic data. While standard variables alone were insufficient—particularly for urinary tract infections—the inclusion of urine analyses, imaging, and point-of-care data significantly enhanced model discrimination and specificity.

The findings highlight that relatively small, clinically relevant additions to routine data can yield substantial improvements in diagnostic performance, especially for infections that are difficult to identify based on symptoms alone. These results support the potential role of multimodal machine learning approaches as decision support tools in the ED, warranting further validation in prospective clinical settings.

## Data Availability

The data analyzed in this study is subject to the following licenses/restrictions: due to Danish laws on personal data, data cannot be shared publicly. To request data, please contact the corresponding author for more information. This organization behind the INDEED study owns the data and can provide access to the final dataset. Requests to access these datasets should be directed to SS, sns@rsyd.dk.
