# Supplementary material for: AI-based clinical prediction model for early infectious disease classification in the emergency department
Source: Front Artif Intell. 2026 Aug 19;9:1894719. doi: 10.3389/frai.2026.1894719 (PMC13533911; doi:10.3389/frai.2026.1894719)
Supplement: Supplementary file 1 [file Data_Sheet_1.PDF]

Supplementary material

Appendix 1 – Standard variables:

Id number
Age
Sex
Weight
Smoker
Triage
Triage score
Respirations frequence
Saturation
Heart rate
Systolic blood pressure
Diastolic blood pressure
Temperature
Glascow Coma Scale (GCS)
Blood samples
Hemoglobin
Leucocytes
Platelets
Neutrophils
Albumin
Creatinine
Urea
Sodium
INR (International Normalized Ratio)
Bilirubin
Glucose
CRP (C-reactive protein)
eGFR (estimated Glomerular Filtration Rate)
Comorbidities
Neurological disease
Dementia
Cerebrovascular disease
Parkinson's disease
Multiple sclerosis
Other neurological disease
Pulmonary disease
Chronic obstructive pulmonary disease (COPD)
Asthma
COVID sequelae
Other pulmonary disease
Endocrine disease
Type 1 diabetes
Type 2 diabetes
Osteoporosis

Other Endocrine disease
Cardiac
Ischemic heart disease
Heart failure
Cardiac arrhythmia
Deep vein thrombosis (DVT)
Pulmonary embolism (PE)
Vascular insufficiency
Other cardiac disease
Nephrological
Chronic kidney disease
Kidney stones
Urinary incontinence
Urinary retention
Other kidney disease
Catheter
Suprapubic catheter (SPC)
Indwelling catheter
Clean Intermittent Catheterization (CIC)
Sterile Intermittent Catheterization (SIC)
Rheumatological
Rheumatoid arthritis
Polymyalgia rheumatica
Gout
Other rheumatological disease
Gastroenterological
Liver disease
Irritable bowel disease
PEG tube
Other gastroenterological disease
Cancer
Lung
Gynecological or gastrointestinal
Hematological
Urinary tract
Esophagus
Ear, nose, or oral cavity
Brain
Breast
Other cancer
Cancer treatment
Chemotherapy
Radiation therapy
Other cancer treatment
Medical record information
Medication
Paracetamol
Non-steroidal anti-inflammatory drugs (NSAIDs)

Opioids
Gabapentin
Other analgesic treatment
Polypharmacy
Clinical findings
Abnormal findings on auscultation
Crepitations on auscultation
Rhonchi on auscultation
Prolonged expiration on auscultation
Dullness on auscultation
Other findings on auscultation
Abdominal tenderness on palpation
Flank pain (left-sided)
Flank pain (right-sided)
Suprapubic pain
Diffuse abdominal pain
Abdominal pain localized elsewhere
Vaccination status
SARS-CoV-2
Pneumococcal vaccine (Pneumovax, Prevenar)
Influenza
No vaccinations
Oxygen supplementation on arrival
Infected

Appendix 2 – Different model variables:

Bio model
Suspected pneumonia diagnosis based on X-ray
Positive x-ray findings
Club cell protein 16 (CC16)
Interleukin-6 (IL-6)
Procalcitonin (PCT)
Soluble urokinase plasminogen activator receptor (suPAR)
Chitinase-3-like protein 1 (YKL-40)
CT model
Suspected pneumonia diagnosis based on X-ray
Positive x-ray findings
Positive lung ultrasound or CT findings
Suspected pneumonia diagnosis based on lung ultrasound or CT
Suspected pneumonia diagnosis based on CT
Positive CT findings
POC model
Suspected pneumonia diagnosis based on X-ray
Positive x-ray findings
Point-of-care PCR: Acinetobacter baumannii
Point-of-care PCR: Enterobacter cloacae

Point-of-care PCR: Escherichia coli
Point-of-care PCR: Haemophilus influenzae
Point-of-care PCR: Klebsiella aerogenes
Point-of-care PCR: Klebsiella oxytoca
Point-of-care PCR: Klebsiella pneumoniae
Point-of-care PCR: Moraxella catarrhalis
Point-of-care PCR: Proteus species
Point-of-care PCR: Pseudomonas aeruginosa
Point-of-care PCR: Serratia marcescens
Point-of-care PCR: Staphylococcus aureus
Point-of-care PCR: Streptococcus agalactiae
Point-of-care PCR: Streptococcus pneumoniae
Point-of-care PCR: Bacterial targets not detected
Point-of-care PCR: Influenza A virus
Point-of-care PCR: Parainfluenza virus
Point-of-care PCR: Coronavirus
Point-of-care PCR: Streptococcus pyogenes
Point-of-care PCR: Chlamydia pneumoniae
Point-of-care PCR: Legionella species
Point-of-care PCR: Mycoplasma pneumoniae
Point-of-care PCR: Influenza B virus
Point-of-care PCR: Adenovirus
Point-of-care PCR: Middle East respiratory syndrome coronavirus (MERS-CoV)
Point-of-care PCR: Human metapneumovirus
Point-of-care PCR: Human rhinovirus/enterovirus
Point-of-care PCR: Respiratory syncytial virus
Point-of-care PCR: Viral targets not detected
Point-of-care PCR resistance marker: mecA/C and MREJ (MRSA)
Point-of-care PCR resistance marker: CTX-M (ESBL)
Point-of-care PCR resistance markers not detected
Urine model
Suspected pneumonia diagnosis based on X-ray
Positive x-ray findings
Urine dipstick: negative
Urine dipstick: glucose
Urine dipstick: ketones
Urine dipstick: blood
Urine dipstick: protein
Urine dipstick: nitrite
Urine dipstick: leukocytes
Urine culture
