# Supplementary material for: AI-based clinical prediction model for early infectious disease classification in the emergency department
Source: Front Artif Intell. 2026 Aug 19;9:1894719. doi: 10.3389/frai.2026.1894719 (PMC13533911; doi:10.3389/frai.2026.1894719)
Supplement: Supplementary file 2 [file Data_Sheet_2.DOCX]

Suplarmentary data

Figure 1:

Figure 1. Calibration curves for the multiclass classification Urine model. The figure presents calibration curves for the three infection disease categories: Pneumonia, UTI, and Other.

Figure 2:

Figure 2. Calibration curves for the multiclass classification Total model. The figure presents calibration curves for the three infection disease categories: Pneumonia, UTI, and Other.
